# A nationwide study of patients with monoclonal gammopathy of undetermined significance with a 10-year follow-up in South Korea

**DOI:** 10.1038/s41598-021-97664-y

**Published:** 2021-09-16

**Authors:** Ka-Won Kang, Ji Eun Song, Byung-Hyun Lee, Min Ji Jeon, Eun Sang Yu, Dae Sik Kim, Se Ryeon Lee, Hwa Jung Sung, Chul Won Choi, Yong Park, Byung Soo Kim

**Affiliations:** 1 Division of Hematology-Oncology, Department of Internal Medicine, Korea University College of Medicine, 73, Goryeodae-ro, Seongbuk-gu, Seoul, 02841 South Korea; 2 Department of Biostatistics, Korea University College of Medicine, Seoul, South Korea

**Keywords:** Diseases, Health care, Medical research, Oncology

## Abstract

In clinical practice, most patients with monoclonal gammopathy of undetermined significance (MGUS) undergo long-term follow-up without disease progression. There is insufficient real-world data about how closely and whether anything other than disease progression should be monitored. Herein, we performed a nationwide study of 470 patients with MGUS with a 10-year follow-up to determine the patterns of disease progression and other comorbidities. During the follow-up period, 158 of 470 patients with MGUS (33.62%) progressed to symptomatic monoclonal gammopathies. Most of these were multiple myeloma (134/470 patients, 28.51%), and those diagnosed within 2 years after diagnosis of MGUS was high. Approximately 30–50% of patients with MGUS had hypertension, diabetes, hyperlipidemia, and osteoarthritis at the time of diagnosis, and these comorbidities were newly developed during the follow-up period in approximately 50% of the remaining patients with MGUS. Approximately 20–40% of patients with MGUS have acute or chronic kidney failure, thyroid disorders, disc disorders, peripheral neuropathy, myocardial infarction, stroke, and heart failure during the follow-up period. Altogether, when MGUS is diagnosed, close follow-up of the possibility of progression to multiple myeloma is required, especially within 2 years after diagnosis; simultaneously, various comorbidities should be considered and monitored during the follow-up of patients with MGUS. Continuous research is needed to establish appropriate follow-up guidelines.

## Introduction

Monoclonal gammopathy of undetermined significance (MGUS) is defined as serum monoclonal protein level < 3 g/dL, bone marrow plasma cells < 10%, and absence of end-organ damage (e.g., hypercalcemia, renal insufficiency, anemia, and bone lesions), or other lymphoproliferative malignancies^1,2^. MGUS is a well-known premalignant phase that can progress to multiple myeloma (MM), Waldenström macroglobulinemia (WM), AL amyloidosis, or other lymphoproliferative disorders at approximately 1% per year^3–5^. Therefore, to detect disease progression, the current guidelines recommend the quantification of monoclonal protein along with monitoring of related symptoms in patients with MGUS at 3–6 month intervals for the first 1–2 years, and then at 6–24 months intervals if stable^4,6–8^.

However, according to data from previous studies, the majority of patients with MGUS do not progress to symptomatic monoclonal gammopathy^9,10^. In addition, patients with MGUS have been shown to have an increased risk of bacterial infections, ischemic heart disease, renal disease, rheumatic diseases, skeletal fractures, and arterial or venous thrombosis^11–16^. Patients with MGUS have a shorter overall survival times compared to the matched control population^17^. Although progression to symptomatic monoclonal gammopathy is an important cause of death in patients with MGUS, it does not entirely explain the shorter overall survival time^18^. Other comorbidities may be associated with shorter overall survival times in patients with MGUS^11,19,20^, it is necessary to reconsider whether it is sufficient to focus solely on disease progression during follow-up and testing according to the recommendations of the guidelines mentioned above.

In this study, we performed a nationwide study with patients diagnosed with MGUS with a follow-up period of 10 years using the Health Insurance Review and Assessment Service (HIRA). This study aimed to determine the occurrence patterns of disease progression and other comorbidities during a 10-year follow-up period in the real world.

## Methods

### Data source

In South Korea, the National Health Insurance (NHI), which is a universal health coverage system, covers approximately 98% of the population^21,22^. Those insured by NHI pay insurance contributions and receive medical services from their health care providers, and then the NHI pays costs based on the claims data. The claims data consist of patients’ diagnosis, treatment, procedures, surgical history, and prescription drugs, and this information is anonymized and provided for healthcare service research in the form of the HIRA database. The data used in this study were extracted from patient information in the HIRA database and were approved by the Institutional Review Board of Korea University Anam Hospital (No. 2020AN0135). Since this study was conducted with anonymized patient data from the HIRA database, the need for informed consent was waived by the institutional review board.

### Patients

In general, the healthcare provider files claims for patient's medical services to the NHI according to the patient's main diagnosis, sub-diagnosis, and rule-out diagnosis. The main diagnosis describes the primary complaint or disease requiring the greatest treatment or examination; the sub-diagnosis was defined as the disease that was present or occurred secondary to the main diagnosis and had an impact on patient care. A rule-out diagnosis was defined as a previously considered disease that was excluded after examination.

To analyze patients with MGUS who had undergone long-term follow-up for 10 years, the claims data for patients diagnosed with MGUS (D472 code of Korea Classification of Disease, 7th edition) as the main diagnosis or sub-diagnosis (excluding rule-out diagnosis) from January 1, 2007, to August 31, 2009, were extracted from January 1, 2007, to August 31, 2019. If a patient progressed to symptomatic monoclonal gammopathy during the follow-up period with MGUS stated as the diagnosis on the first claim data within the recruitment period of this study, it is possible that the two claim codes (MGUS as the main diagnosis and symptomatic monoclonal gammopathy as sub-diagnosis, or vice versa) could be claimed on the same date. These patients were not suitable for this study; therefore, patients with MM, plasma cell leukemia (PCL), plasmacytoma, WM, amyloidosis, and any lymphoproliferative diseases or hematologic malignancies at the date of diagnosis of MGUS were excluded (Supplementary Table 1).

This study aimed to determine the prevalence and occurrence patterns of disease progression and other comorbidities of MGUS in the real world. Therefore, all patients who met the inclusion criteria were presented without limitations, such as age.

### Clinical endpoints

We aimed to confirm the prevalence of MGUS in South Korea and to determine the occurrence patterns of disease progression and other comorbidities during the 10-year follow-up period.

**Figure 1:**
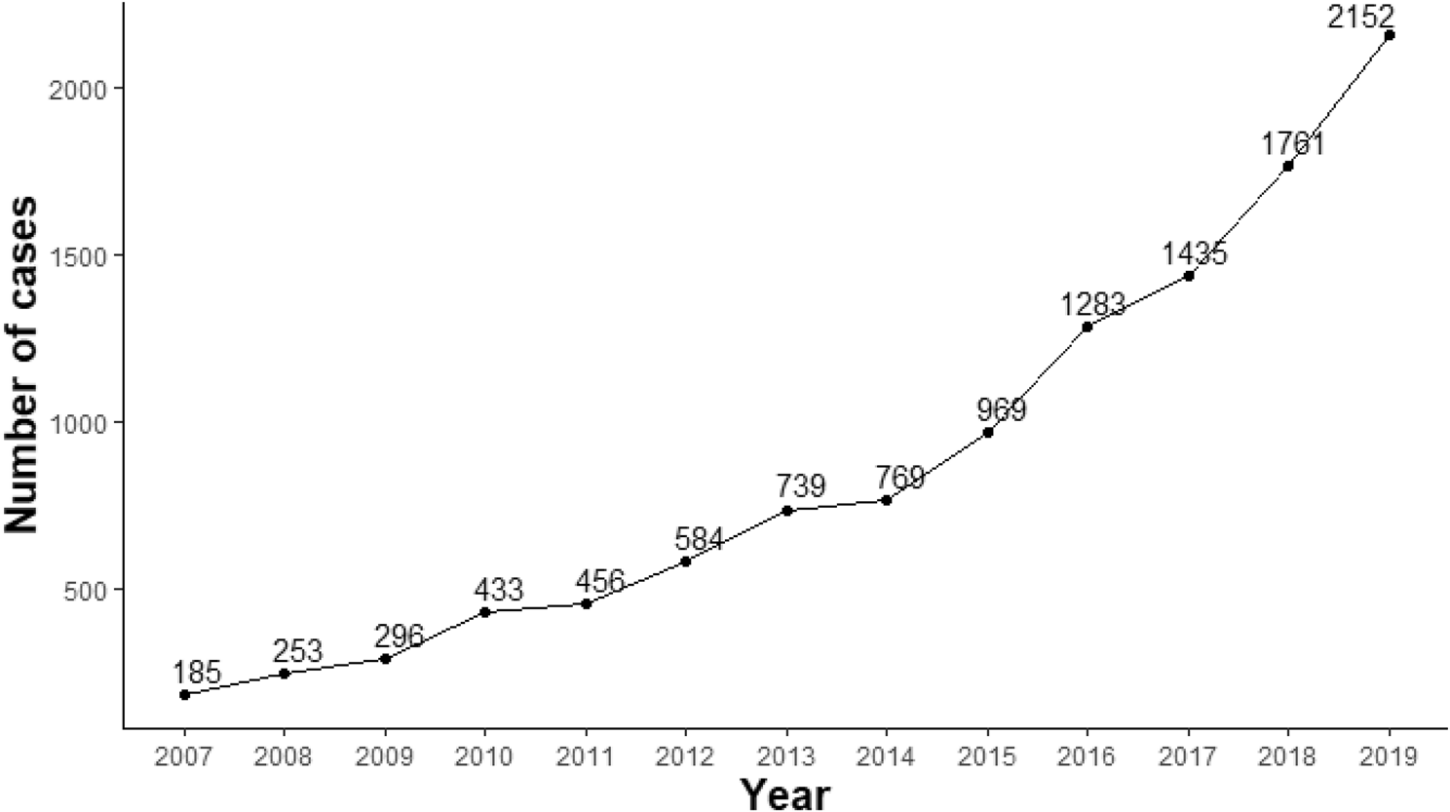
The number of patients with MGUS in South Korea according to the year. The data for the number of patients with MGUS in 2007 and 2008 were derived from the HIRA database of this study, and the data after 2009 were derived from the public data provided by HIRA.

The prevalence of MGUS was calculated by dividing the number of patients with MGUS by the mid-year population (Table 1 and Supplementary Table 2). The HIRA only provides claim data for the patient population that the researcher intends to study (HIRA policy due to database serving capacity limitations). Instead, information on the number of patients with specific diseases (excluding details about that population) is disclosed to the general public as public data. In this study, we analyzed the claims data extracted from January 1, 2007, to August 31, 2019, for patients who were diagnosed with MGUS from January 1, 2007, to August 31, 2009. To present the number of patients with MGUS from 2007 to 2019, the number of patients with MGUS in 2007 and 2008 was derived from the HIRA database, and the data after 2009 were derived from the public data provided by HIRA (Fig. 1).

**Table 1:** Prevalence of MGUS in South Korea between January 1, 2007, and August 31, 2009.

Year	Among all ages				Among those aged 50 years or older																						
	A	B	A/B	Prevalence rate per 100,000 population	A	B	A/B	Prevalence rate per 100,000 population																			
A. Prevalence rate per 100,000 population																											
2007	185	50,144,604	0.0000037	0.37	149	13,383,794	0.0000111	1.11																			
2008	253	50,498,196	0.0000050	0.50	213	14,049,216	0.0000152	1.52																			
2009*	255	50,833,594	0.0000050	0.50	224	14,756,857	0.0000152	1.52																			

Age	2007									2008									2009*								
	Male			Female			Total			Male			Female			Total			Male			Female			Total		
	A	B	A/B	A	B	A/B	A	B	A/B	A	B	A/B	A	B	A/B	A	B	A/B	A	B	A/B	A	B	A/B	A	B	A/B
B. Prevalence by age group																											
0—9 years	0	2,791,124	━	0	2,569,569	━	0	5,360,693	━	0	2,695,506	━	1	2,488,333	0.0000000	1	5,183,839	0	0	2,600,690	━	0	2,409,226	━	0	5,009,916	━
10 -19 years	0	3,589,335	━	1	3,193,224	0.0000003	1	6,782,559	0.0000001	1	3,605,985	0.0000003	0	3,211,512	━	1	6,817,497	0.0000001	0	3,603,701	━	0	3,214,585	━	0	6,818,286	━
20–29 years	0	3,812,687	━	1	3,592,012	0.0000003	1	7,404,699	0.0000001	0	3,751,232	━	1	3,522,600	0.0000003	1	7,273,832	0.0000001	1	3,682,227	0.0000003	1	3,444,724	0.0000003	2	7,126,950	0.0000003
30–39 years	3	4,431,071	0.0000007	9	4,283,377	0.0000021	12	8,714,448	0.0000014	1	4,366,438	0.0000002	9	4,204,411	0.0000021	10	8,570,849	0.0000012	2	4,295,222	0.0000005	5	4,125,692	0.0000012	7	8,420,914	0.0000008
40–49 years	5	4,333,766	0.0000012	17	4,164,648	0.0000041	22	8,498,413	0.0000026	13	4,377,870	0.0000030	12	4,225,095	0.0000028	25	8,602,965	0.0000029	7	4,424,693	0.0000016	13	4,275,979	0.0000030	20	8,700,672	0.0000023
50–59 years	27	2,851,682	0.0000095	16	2,850,028	0.0000056	43	5,701,710	0.0000075	23	3,002,131	0.0000077	20	2,989,519	0.0000067	43	5,991,650	0.0000072	26	3,170,224	0.0000082	25	3,154,102	0.0000079	51	6,324,325	0.0000081
60–69 years	28	1,769,337	0.0000158	18	2,020,062	0.0000089	46	3,789,399	0.0000121	53	1,837,226	0.0000288	30	2,072,524	0.0000145	83	3,909,750	0.0000212	51	1,894,727	0.0000269	26	2,111,417	0.0000123	77	4,006,144	0.0000192
70–79 years	25	832,057	0.0000300	19	1,313,137	0.0000145	44	2,145,193	0.0000205	48	893,794	0.0000537	25	1,375,532	0.0000182	73	2,269,326	0.0000322	44	961,242	0.0000458	31	1,443,998	0.0000215	75	2,405,240	0.0000312
80–89 years	8	482,118	0.0000166	5	1,187,200	0.0000042	13	1,669,317	0.0000078	8	515,639	0.0000155	7	1,279,110	0.0000055	15	1,794,749	0.0000084	12	554,120	0.0000217	9	1,377,303	0.0000065	21	1,931,423	0.0000109
90–99 years	3	15,705	0.0001910	0	60,400	━	3	76,105	0.0000394	1	16,991	0.0000589	0	64,511	━	1	81,501	0.0000123	2	18,560	0.0001078	0	68,699	━	2	87,259	0.0000229
≥ 100 years	0	216	━	0	1,855	━	0	2,071	━		243	━	0	1,999	━	0	2,242	━	0	308	━	0	2,159	━	0	2,467	━
Total	99	24,909,095	0.0000040	86	25,235,509	0.0000034	185	50,144,604	0.0000037	148	25,063,053	0.0000059	105	25,435,144	0.0000041	253	50,498,196	0.0000050	145	25,205,712	0.0000058	110	25,627,882	0.0000043	255	50,833,594	0.0000050

A represents the number of patients with MGUS during the given year. B represents the mid-year population of the given year according to Korean Statistical Information Service survey results (Supplementary Table 2). * Eight months of data from January 1, 2009, and August 31, 2009, were summarized.

Disease progression was defined as the occurrence of MM, PCL, plasmacytoma, WM, amyloidosis, and any lymphoproliferative disease or hematologic malignancies after the date of diagnosis of MGUS. The incidence of disease progression was presented as the number of new patients in the year normalized to the number of patients with MGUS in this study. The prevalence was presented as the number of total patients in the year normalized to the number of patients MGUS in this study.

Comorbidities included chronic diseases known to be common in South Korea^23^ or diseases commonly associated with patients with MGUS in existing studies^11–13,16^. For solid malignancies, the seven most common cancers in South Korea were selected^24^. The prevalence of comorbidities at the diagnosis of MGUS was defined as comorbidities diagnosed from January 1, 2007, to the date of MGUS diagnosis. Newly developed comorbidities were defined as newly developed diseases in patients with MGUS who did not have any comorbidities at the time of MGUS diagnosis. As mentioned above, the HIRA provided claims data for patients who were diagnosed with MGUS from January 1, 2007, to August 31, 2009. To determine the prevalence of comorbidities in patients with MGUS compared to the entire population, the prevalence data for each comorbidity in the entire population (determined from public data from the HIRA database) were presented as reference data. The prevalence calculation method for each comorbidity in the entire population is presented as a figure legend in Supplementary Figure 1. The incidence and prevalence of newly developed comorbidities were presented as the number of new patients in the year and total number of patients in the year, respectively.

### Statistical analysis

Categorical data are presented as frequencies and percentages. Continuous data are described as the mean with standard deviation. Statistical analyses were performed using SAS v9.4 (SAS Institute Inc., Cary, NC, USA) and R Statistical Software v3.3.3 (Foundation for Statistical Computing, Vienna, Austria).

## Results

### Patients with MGUS in South Korea

Between January 1, 2007, and August 31, 2009, 643 patients with MGUS were identified. A total of 470 patients were analyzed in this study, after excluding patients with MM, PCL, plasmacytoma, WM, amyloidosis, lymphoproliferative diseases, and hematologic malignancies at the time of diagnosis of MGUS (Supplementary Table 1). Among 470 patients with MGUS, bone marrow examination, spinal computed tomography (CT), or spinal magnetic resonance imaging (MRI) was performed within 30 days before the date of diagnosis with MGUS in 20.2% (95/470), 1.5% (7/470), and 0.9% (4/470) of cases, respectively. None of the patients with MGUS underwent positron emission tomography.

The prevalence rate per 100,000 people in South Korea between January 1, 2007, and August 31, 2009, was 0.37–0.50 overall and 1.11–1.52 in those aged 50 years or older. The prevalence of MGUS was higher in individuals aged 50 years and older and in men (Table 1). The prevalence has been shown to increase annually, and from around 2015, the trend of increase has become steeper (Fig. 1).

### The occurrence patterns of disease progression during the follow-up period

Disease progression after the date of MGUS diagnosis during the follow-up period is summarized in Table 2. MM occurred in 28.51% (134 patients) of the 470 patients with MGUS. PCL, extramedullary plasmacytoma, and solitary plasmacytoma occurred in 0.43% (2 patients), 0.64% (3 patients), and 0.43% (2 patients), respectively. WM and amyloidosis occurred in 1.7% (7 patients) and 2.5% (10 patients), respectively. Lymphoma occurred in 4.68% (22 patients) and leukemia in 3.62% (17 patients).

**Table 2:** Disease progression after the date of diagnosis of MGUS during the follow-up period.

Type of disease progression	Number (%)
C90.0 Multiple myeloma	134 (28.51)
C90.1 Plasma cell leukemia	2 (0.43)
C90.2 Extramedullary plasmacytoma	3 (0.64)
C90.3 Solitary plasmacytoma	2 (0.43)
C88.0 Waldenström macroglobulinemia	7 (1.49)
E85.3, E85.4, E85.8, E85.9 Amyloidosis^†^	10 (2.13)
C81 Hodgkin lymphoma^‡^	1 (0.21)
C82 Follicular lymphoma^‡^	1 (0.21)
C83 Non-follicular lymphoma^‡^	9 (1.91)
C84 Mature T/NK-cell lymphomas^‡^	0 (0.00)
C85 Other and unspecified types of non-Hodgkin lymphoma^‡^	11 (2.34)
C86 Other specified types of T/NK-cell lymphoma^‡^	0 (0.00)
C88.4 Extranodal marginal zone B-cell lymphoma of mucosa-associated lymphoid tissue [MALT-lymphoma]	0 (0.00)
C91 Lymphoid leukemia^‡^	7 (1.49)
C92 Myeloid leukemia^‡^	7 (1.49)
C93 Monocytic leukemia^‡^	0 (0.00)
C94 Other leukemias of specified cell type^‡^	1 (0.21)
C95 Leukemia of unspecified cell type^‡^	2 (0.43)
C96 Other and unspecified malignant neoplasms of lymphoid, hematopoietic and related tissue^‡^	2 (0.43)
D89.1 Cryoglobulinemia	2 (0.43)

† Only secondary amyloidosis was selected. ‡ This entry includes all sub-codes.

**Figure 2:**
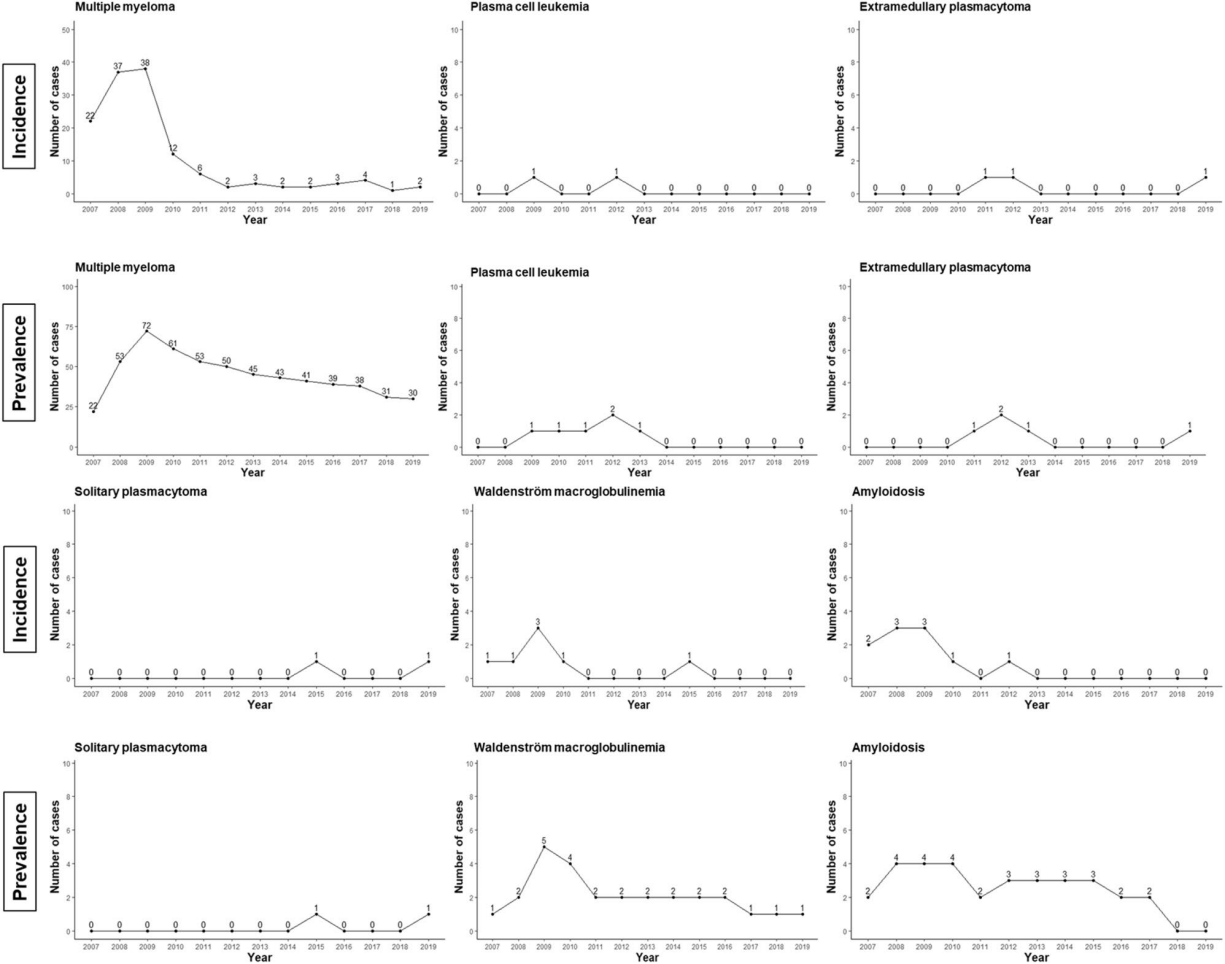
The occurrence patterns of disease progression during the follow-up period. The incidence of disease progression was presented as the number of new patients in the year in patients with MGUS in this study. The prevalence was presented as the number of total patients in the year in patients with MGUS in this study.

The occurrence patterns of disease progression during the follow-up period of MM, PCL, extramedullary plasmacytoma, solitary plasmacytoma, WM, and amyloidosis are shown in Fig. 2. In the case of MM, WM, and amyloidosis, the probability of occurrence within 2 years of initial diagnosis of MGUS tended to be high. PCL, extramedullary plasmacytoma, and solitary plasmacytoma showed a tendency to occur sporadically throughout the follow-up period. In patients who progressed to MM, the median duration from the date of diagnosis of MGUS to the date of diagnosis of MM was 2.5 months (range: 0.1–147.4 months) (Supplementary Figure 2).

### The occurrence patterns of comorbidities during the follow-up period

**Figure 3:**
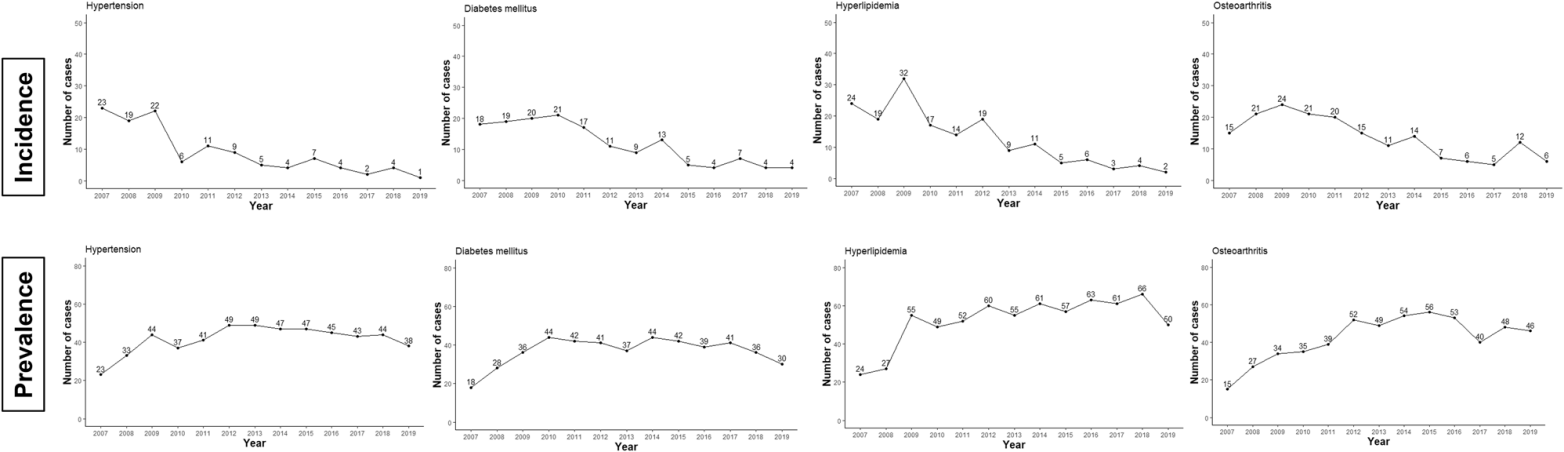
The occurrence patterns of comorbidities during the follow-up period. The incidence and prevalence of newly developed comorbidities were presented as the number of new patients in the year and total number of patients in the year, respectively.

The prevalence of comorbidities and newly developed comorbidities during the follow-up period are summarized in Tables 3 and 4. Population-wide prevalence data for each comorbidity are presented as reference data in Table 3. The prevalence calculation method for each comorbidity is presented as a figure legend in Supplementary Figure 1. At the time of diagnosis of MGUS, 54.04% (254/470 patients), 40.64% (191/470 patients), 42.55% (200/470 patients), and 29.57% (139/470 patients) had accompanying hypertension (HTN), diabetes mellitus (DM), hyperlipidemia (HLD), and osteoarthritis (OA), respectively. During the follow-up period, HTN, DM, HLD, and OA were newly developed (i.e., not present at the time of diagnosis of MGUS) in 54.17% (117/216 patients), 54.48% (152/279 patients), 61.11% (165/270 patients), and 53.47% (177/331 patients) of patients, respectively. The occurrence pattern of these comorbidities appeared to occur within 5 years after the date of MGUS diagnosis (Fig. 3). Solid malignancies occurred in 5% of the patients during the follow-up period.

**Table 3:** Prevalence of comorbidities at the time of MGUS diagnosis.

Baseline characteristics	Total number of the patients with MGUS (%)	Number of patients with MGUS aged > 50 years (%)	Prevalence data extracted from the public database of HIRA in 2010*			
			Total number of patients	Overall prevalence (%)^∫^	Number of patients aged over 50 years	Prevalence in those aged over 50 years (%)^∬^
**Age at diagnosis, year**						
n	470					
Mean (standard deviation)	63.47 (13.60)					
Median (min, max)	66 (3, 91)					
**Sex, n (%)**						
Male	260 (55.32)					
Female	210 (44.68)					
**Chronic disease, n (%)**						
**Hypertension‡**	254 (54.04)	236 (50.21)				
I10 Essential (primary) hypertension			4,813,897	9.4130	4,466,593	28.7713
**Diabetes mellitus‡**	191 (40.64)					
E10 Type 1 diabetes mellitus	21 (4.47)	20 (4.26)	97,573	0.1908	77,087	0.4966
E11 Type 2 diabetes mellitus	161 (34.26)	150 (31.91)	1,719,221	3.3617	1,535,310	9.8896
E12 Malnutrition-related diabetes mellitus	3 (0.64)	3 (0.64)	4,883	0.0095	4,671	0.0301
E13 Other specified diabetes mellitus	12 (2.55)	11 (2.34)	59,665	0.1167	51,284	0.3303
E14 Unspecified diabetes mellitus	85 (18.09)	80 (17.02)	371,413	0.7263	323,378	2.0830
**Hyperlipidemia**	200 (42.55)	184 (39.15)				
E78.0 Pure hypercholesterolemia	83 (17.45)	77 (16.38)	246,413	0.4818	188,604	1.2149
E78.1 Pure hyperglyceridemia	22 (4.68)	20 (4.26)	37,270	0.0729	22,841	0.1471
E78.2 Mixed hyperlipidemia	55 (11.70)	51 (10.85)	228,735	0.4473	165,505	1.0661
E78.3 Hyperchylomicronemia	0	0	794	0.0016	571	0.0037
E78.4 Other hyperlipidemia	31 (6.60)	30 (6.38)	133,471	0.2610	99,484	0.6408
E78.5 Hyperlipidemia, unspecified	110 (23.40)	104 (22.13)	440,904	0.8621	316,286	2.0373
**Arteriosclerosis**	32 (6.81)	32 (6.81)				
I70 Atherosclerosis ‡	29 (6.17)	29 (6.17)	62,392	0.1220	55,513	0.3576
I67.2 Cerebral atherosclerosis	5 (1.06)	5 (1.06)	19,636	0.0384	18,215	0.1173
I25.0 Atherosclerotic cardiovascular disease, so described	0	0	3059	0.0060	2,758	0.0178
**Disorders of thyroid‡**	81 (17.23)	70 (14.89)				
E02 Subclinical iodine-deficiency hypothyroidism	0	0	3,694	0.0072	1,601	0.0103
E03 Other hypothyroidism	46 (9.79)	38 (8.09)	314,847	0.6156	147,492	0.9501
E05 Thyrotoxicosis[hyperthyroidism]	41 (8.72)	37 (7.87)	241,184	0.4716	90,530	0.5831
E06 Thyroiditis	7 (1.49)	6 (1.28)	106,382	0.2080	41,098	0.2647
**Peripheral neuropathy**	52 (11.06)	46 (9.79)				
G61.8 Other inflammatory polyneuropathies	6 (1.28)	6 (1.28)	472	0.0009	311	0.0020
G61.9 Inflammatory polyneuropathy, unspecified	4 (0.85)	4 (0.85)	453	0.0009	344	0.0022
G62.8 Other specified polyneuropathies	13 (2.77)	11 (2.34)	3,280	0.0064	2,227	0.0143
G62.9 Polyneuropathy, unspecified	36 (7.66)	32 (6.81)	27,429	0.0536	19,776	0.1274
G64 Other disorders of peripheral nervous system‡	9 (1.91)	8 (1.7)	22,393	0.0438	15,494	0.0998
**Skeletal-related events, n (%)**						
**Osteoarthritis**	139 (29.57)	136 (28.94)				
M15 Polyarthrosis‡	31 (6.60)	31 (6.6)	283,023	0.5534	241,338	1.5546
M16.0, M16.1, M16.9 Coxarthrosis [arthrosis of hip]	16 (3.40)	16 (3.4)				
M16.0 Primary coxarthrosis, bilateral	6 (1.28)	6 (1.28)	18,008	0.0352	15,169	0.0977
M16.1 Other primary coxarthrosis	2 (0.43)	2 (0.43)	16,964	0.0332	13,518	0.0871
M16.9 Coxarthrosis, unspecified	8 (1.70)	8 (1.7)	44,723	0.0875	34,144	0.2199
M17.0, M17.1, M17.9 Gonarthrosis[arthrosis of knee]	93 (19.79)	93 (19.79)				
M17.0 Primary gonarthrosis, bilateral	48 (10.21)	48 (10.21)	1,202,930	2.3522	1,117,628	7.1991
M17.1 Other primary gonarthrosis	38 (8.09)	38 (8.09)	671,992	1.3140	598,253	3.8536
M17.9 Gonarthrosis, unspecified	39 (8.30)	39 (8.3)	717,457	1.4029	620,633	3.9978
M18.0, M18.1, M18.9 Arthrosis of first carpometacarpal joint	0	0				
M18.0 Primary arthrosis of first carpometacarpal joints, bilateral	0	0	3,057	0.0060	2,287	0.0147
M18.1 Other primary arthrosis of first carpome- tacarpal joint	0	0	2,253	0.0044	1,498	0.0096
M18.9 Arthrosis of first carpometacarpal joint, unspecified	0	0	4,397	0.0086	3,270	0.0211
M19 Other arthrosis‡	57 (12.13)	54 (11.49)	626,012	1.2241	465,302	2.9972
**Rheumatoid arthritis‡**	57 (12.13)	48 (10.21)				
M05 Seropositive rheumatoid arthritis	14 (2.98)	13 (2.77)	72,569	0.1419	56,008	0.3608
M06 Other rheumatoid arthritis	47 (10.00)	39 (8.3)	225,998	0.4419	156,392	1.0074
**Disc disorder‡**	76 (16.17)	68 (14.47)				
M50 Cervical disc disorders	36 (7.66)	30 (6.38)	694,974	1.3589	417,861	2.6916
M51 Other intervertebral disc disorders	53 (11.28)	49 (10.43)	1,614,820	3.1576	982,281	6.3273
**Osteoporosis**	32 (6.81)	30 (6.38)				
M80.5, M80.8, M80.9 Osteoporosis with pathological fracture	4 (0.85)	4 (0.85)				
M80.5 Idiopathic osteoporosis with pathological fracture	0	0	4,508	0.0088	4,483	0.0289
M80.8 Other osteoporosis with pathological fracture	1 (0.21)	1 (0.21)	16,524	0.0323	16,528	0.1065
M80.9 Unspecified osteoporosis with pathological fracture	3 (0.64)	3 (0.64)	17,738	0.0347	17,632	0.1136
M81.5, M81.8, M81.9 Osteoporosis without pathological fracture	29 (6.17)	27 (5.74)				
M81.5 Idiopathic osteoporosis	1 (0.21)	1 (0.21)	35,336	0.0691	32,440	0.2090
M81.8 Other osteoporosis	5 (1.06)	5 (1.06)	192,560	0.3765	182,604	1.1762
M81.9 Osteoporosis, unspecified	26 (5.53)	24 (5.11)	259,860	0.5081	242,950	1.5649
**Venous thrombosis, n (%)**						
I80 Phlebitis and thrombophlebitis	6 (1.28)	6 (1.28)				
I80.0 Phlebitis and thrombophlebitis of superficial vessels of lower extremities	0	0	1,595	0.0031	797	0.0051
I80.1 Phlebitis and thrombophlebitis of femoral vein	0	0	496	0.0010	294	0.0019
I80.2 Phlebitis and thrombophlebitis of other deep vessels of lower extremities	3 (0.64)	3 (0.64)	8,307	0.0162	6,118	0.0394
I80.3 Phlebitis and thrombophlebitis of lower extremities, unspecified	1 (0.21)	1 (0.21)	1,761	0.0034	1,040	0.0067
I80.8 Phlebitis and thrombophlebitis of other sites	1 (0.21)	1 (0.21)	2,621	0.0051	1,129	0.0073
I80.9 Phlebitis and thrombophlebitis of unspecified site	1 (0.21)	1 (0.21)	2,555	0.0050	1,239	0.0080
I81 Portal vein thrombosis‡	0	0	358	0.0007	161	0.0010
I82 Other venous embolism and thrombosis	3 (0.64)	3 (0.64)				
I82.0 Budd-Chiari syndrome	0	0	214	0.0004	134	0.0009
I82.1 Thrombophlebitis migrans	0	0	60	0.0001	23	0.0001
I82.2 Embolism and thrombosis of vena cava	0	0	166	0.0003	117	0.0008
I82.3 Embolism and thrombosis of renal vein	0	0	90	0.0002	50	0.0003
I82.8 Embolism and thrombosis of other specified veins	2 (0.43)	2 (0.43)	1,833	0.0036	1,271	0.0082
I82.9 Embolism and thrombosis of unspecified vein	1 (0.21)	1 (0.21)	2,759	0.0054	2,063	0.0133
G08 Intracranial and intraspinal phlebitis and thrombophlebitis‡	0	0	208	0.0004	102	0.0007
G95.1 Vascular myelopathies	8 (1.70)	8 (1.7)	55	0.0001	34	0.0002
K55.0 Acute vascular disorders of intestine	0	0	1,438	0.0028	1,026	0.0066
K55.1 Chronic vascular disorders of intestine	0	0	484	0.0009	310	0.0020
**Arterial thrombosis, n (%)**						
Myocardial infarction‡	65 (13.83)	59 (12.55)				
I21 Acute myocardial infarction	20 (4.26)	18 (3.83)	66,572	0.1302	60,760	0.3914
I22 Subsequent myocardial infarction	1 (0.21)	0	2,087	0.0041	1,958	0.0126
I23 Certain current complications following acute myocardial infarction	0	0	674	0.0013	636	0.0041
I24 Other acute ischemic heart diseases	7 (1.49)	7 (1.49)	8,610	0.0168	6,796	0.0438
I25 Chronic ischemic heart disease	46 (9.79)	43 (9.15)	148,848	0.2911	140,131	0.9026
I26 Pulmonary embolism‡	5 (1.06)	3 (0.64)	6,985	0.0137	6,290	0.0405
Stroke‡	55 (11.70)	52 (11.06)				
I63.0 Cerebral infarction due to thrombosis of precerebral arteries	1 (0.21)	1 (0.21)	19,377	0.0379	18,874	0.1216
I63.1 Cerebral infarction due to embolism of precerebral arteries	1 (0.21)	1 (0.21)	4,806	0.0094	4,661	0.0300
I63.2 Cerebral infarction due to unspecified occlusion or stenosis of precerebral arteries	2 0.43)	2 (0.43)	8,044	0.0157	7,688	0.0495
I63.3 Cerebral infarction due to thrombosis of cerebral arteries	5 (1.06)	5 (1.06)	47,969	0.0938	46,933	0.3023
I63.4 Cerebral infarction due to embolism of cerebral arteries	0	0				
I63.5 Cerebral infarction due to unspecified occlusion or stenosis of cerebral arteries	8 (1.70)	8 (1.7)	29,994	0.0586	28,740	0.1851
I63.6 Cerebral infarction due to cerebral venous thrombosis, nonpyogenic	0	0	1,071	0.0021	966	0.0062
I63.8 Other cerebral infarction	8 (1.70)	8 (1.7)	88,088	0.1722	84,596	0.5449
I63.9 Cerebral infarction, unspecified	41 (8.72)	38 (8.09)	279,545	0.5466	270,495	1.7424
I67.6 Nonpyogenic thrombosis of intracranial venous system	0	0	130	0.0003	74	0.0005
**Acute renal failure‡, n (%)**						
N17 Acute renal failure	41 (8.72)	39 (8.3)	14,205	0.0278	11,073	0.0713
N19 Unspecified kidney failure	15 (3.19)	13 (2.77)	7,172	0.0140	5,422	0.0349
**Chronic renal failure‡, n (%)**						
N18.1 Chronic kidney disease, stage 1	0	0	1,899	0.0037	1,382	0.0089
N18.2 Chronic kidney disease, stage 2	0	0	4,239	0.0083	3,402	0.0219
N18.3 Chronic kidney disease, stage 3	0	0	13,349	0.0261	11,714	0.0755
N18.4 Chronic kidney disease, stage 4	0	0	9,513	0.0186	8,319	0.0536
N18.5 Chronic kidney disease, stage 5	0	0	46,978	0.0919	38,412	0.2474
N18.9 Chronic kidney disease, unspecified	58 (12.34)	53 (11.28)	66,530	0.1301	55,966	0.3605
**Heart failure‡, n (%)**						
I50 Heart failure	53 (11.28)	52 (11.06)	99,708	0.1950	96,162	0.6194
**Malignancy‡, n (%)**						
C16, Malignant neoplasm of stomach	8 (1.70)	8 (1.7)	134,958	0.2639	122,872	0.7915
C18, C19, C20 Colorectal cancer	0	0				
C18 Malignant neoplasm of colon	0	0	65,102	0.1273	62,043	0.3996
C19 Malignant neoplasm of rectosigmoid junction	0	0	8,943	0.0175	8,190	0.0528
C20 Malignant neoplasm of rectum	0	0	45,311	0.0886	41,426	0.2668
C33, C34 Lung cancer	12 (2.55)	12 (2.55)				
C33 Malignant neoplasm of trachea	0	0	256	0.0005	184	0.0012
C34 Malignant neoplasm of bronchus and lung	12 (2.55)	12 (2.55)	54,974	0.1075	54,890	0.3536
C73 Malignant neoplasm of thyroid gland	3 (0.64)	3 (0.64)	167,683	0.3279	89,159	0.5743
C50 Malignant neoplasm of breast	2 (0.43)	1 (0.21)	97,008	0.1897	64,237	0.4138
C22 Malignant neoplasm of liver and intrahepatic bile ducts	16 (3.40)	16 (3.4)	54,467	0.1065	50,835	0.3275
C61 Malignant neoplasm of prostate	11 (2.34)	11 (2.34)	35,688	0.0698	36,902	0.2377

The prevalence of comorbidities at the of MGUS time diagnosis was defined as comorbidities diagnosed from January 1, 2007 to the date for diagnosis of MGUS. *The prevalence data for each comorbidity in the entire population was provided by the public HIRA database, presented as reference data. The prevalence calculation method of each comorbidity in the entire population is presented as a figure legend in Supplementary Figure 1. ∫The overall prevalence was calculated by the following formula: (the total number of patients diagnosed with corresponding disease during the year/the mid-year population) × 100. ∬ The prevalence among those aged > 50 years was calculated by the following formula: (the number of patients > 50 years diagnosed with the corresponding disease during the year/the mid-year population > 50 years) × 100. ‡ This entry includes all sub-codes.

**Table 4:** Newly developed comorbidities during the follow-up period.

Comorbidities	n (%)
**Chronic disease, n (%)**	
**Hypertension‡**	117 (54.17)
I10 Essential (primary) hypertension	
**Diabetes mellitus‡**	152 (54.48)
E10 Type 1 diabetes mellitus	39
E11 Type 2 diabetes mellitus	141
E12 Malnutrition-related diabetes mellitus	0
E13 Other specified diabetes mellitus	30
E14 Unspecified diabetes mellitus	114
**Hyperlipidemia**	165 (61.11)
E78.0 Pure hypercholesterolemia	107
E78.1 Pure hyperglyceridemia	30
E78.2 Mixed hyperlipidemia	84
E78.3 Hyperchylomicronemia	0
E78.4 Other hyperlipidemia	75
E78.5 Hyperlipidemia, unspecified	193
**Arteriosclerosis**	75 (17.12)
I70 Atherosclerosis ‡	60
I67.2 Cerebral atherosclerosis	15
I25.0 Atherosclerotic cardiovascular disease, so described	4
**Disorders of thyroid‡**	101 (25.96)
E02 Subclinical iodine-deficiency hypothyroidism	4
E03 Other hypothyroidism	78
E05 Thyrotoxicosis[hyperthyroidism]	42
E06 Thyroiditis	33
**Peripheral neuropathy**	64 (15.31)
G61.8 Other inflammatory polyneuropathies	3
G61.9 Inflammatory polyneuropathy, unspecified	3
G62.8 Other specified polyneuropathies	8
G62.9 Polyneuropathy, unspecified	43
G64 Other disorders of peripheral nervous system‡	35
**Skeletal-related events, n (%)**	
**Osteoarthritis**	177 (53.47)
M15 Polyarthrosis‡	73
M16.0, M16.1, M16.9 Coxarthrosis [arthrosis of hip]	18
M17.0, M17.1, M17.9 Gonarthrosis[arthrosis of knee]	133
M18.0, M18.1, M18.9 Arthrosis of first carpometacarpal joint	4
M19 Other arthrosis‡	145
**Rheumatoid arthritis‡**	64 (15.50)
M05 Seropositive rheumatoid arthritis	16
M06 Other rheumatoid arthritis	68
**Disc disorder‡**	134 (34.01)
M50 Cervical disc disorders	72
M51 Other intervertebral disc disorders	126
**Osteoporosis**	30 (6.85)
M80.5, M80.8, M80.9 Osteoporosis with pathological fracture	8
M81.5, M81.8, M81.9 Osteoporosis without pathological fracture	26
**Venous thrombosis, n (%)**	
I80 Phlebitis and thrombophlebitis	27 (5.82)
I80.0 Phlebitis and thrombophlebitis of superficial vessels of lower extremities	2
I80.1 Phlebitis and thrombophlebitis of femoral vein	0
I80.2 Phlebitis and thrombophlebitis of other deep vessels of lower extremities	12
I80.3 Phlebitis and thrombophlebitis of lower extremities, unspecified	1
I80.8 Phlebitis and thrombophlebitis of other sites	8
I80.9 Phlebitis and thrombophlebitis of unspecified site	11
I81 Portal vein thrombosis‡	2 (0.43)
I82 Other venous embolism and thrombosis	21 (4.50)
I82.0 Budd-Chiari syndrome	0
I82.1 Thrombophlebitis migrans	0
I82.2 Embolism and thrombosis of vena cava	0
I82.3 Embolism and thrombosis of renal vein	1
I82.8 Embolism and thrombosis of other specified veins	9
I82.9 Embolism and thrombosis of unspecified vein	14
G08 Intracranial and intraspinal phlebitis and thrombophlebitis‡	0
G95.1 Vascular myelopathies	8 (1.73)
K55.0 Acute vascular disorders of intestine	1 (0.21)
K55.1 Chronic vascular disorders of intestine	0
**Arterial thrombosis, n (%)**	
Myocardial infarction‡	73 (18.02)
I21 Acute myocardial infarction	38
I22 Subsequent myocardial infarction	1
I23 Certain current complications following acute myocardial infarction	2
I24 Other acute ischemic heart diseases	7
I25 Chronic ischemic heart disease	50
I26 Pulmonary embolism‡	8 (1.72)
Stroke‡	67 (16.14)
I63.0 Cerebral infarction due to thrombosis of precerebral arteries	8
I63.1 Cerebral infarction due to embolism of precerebral arteries	2
I63.2 Cerebral infarction due to unspecified occlusion or stenosis of precerebral arteries	8
I63.3 Cerebral infarction due to thrombosis of cerebral arteries	8
I63.4 Cerebral infarction due to embolism of cerebral arteries	3
I63.5 Cerebral infarction due to unspecified occlusion or stenosis of cerebral arteries	7
I63.6 Cerebral infarction due to cerebral venous thrombosis, nonpyogenic	2
I63.8 Other cerebral infarction	25
I63.9 Cerebral infarction, unspecified	55
I67.6 Nonpyogenic thrombosis of intracranial venous system	0
**Acute renal failure, n (%)**	
N17 Acute renal failure	69 (16.08)
N19 Unspecified kidney failure	34 (7.47)
**Chronic renal failrue, n (%)**	
N18.1 Chronic kidney disease, stage 1	12 (2.55)
N18.2 Chronic kidney disease, stage 2	5 (1.06)
N18.3 Chronic kidney disease, stage 3	23 (4.89)
N18.4 Chronic kidney disease, stage 4	24 (5.11)
N18.5 Chronic kidney disease, stage 5	52 (11.06)
N18.9 Chronic kidney disease, unspecified	68 (16.50)
**Heart failure, n (%)**	
I50 Heart failure	89 (21.34)
**Malignancy, n (%)**	
C16, Malignant neoplasm of stomach	15 (3.25)
C18, C19, C20 Colorectal cancer	0
C33, C34 Lung cancer	12 (2.55)
C73 Malignant neoplasm of thyroid gland	9 (1.93)
C50 Malignant neoplasm of breast	2 (0.43)
C22 Malignant neoplasm of liver and intrahepatic bile ducts	16 (3.40)
C61 Malignant neoplasm of prostate	13 (2.83)

Newly developed comorbidities were defined as newly developed diseases in patients with MGUS who did not have the indicated comorbidity at the time of MGUS diagnosis. ‡ This entry includes all sub-codes.

## Discussion

In this study, the prevalence rate of MGUS was 1.11–1.52 in individuals aged 50 years or older per 100,000 population in South Korea between January 1, 2007, and August 31, 2009, and this has been gradually increasing. During a 10-year follow-up of patients with MGUS, 158 of 470 patients (33.62%) progressed to symptomatic monoclonal gammopathies. Most of these were MM (134/470 patients, 28.51%), and the number of cases diagnosed within 2 years after the diagnosis of MGUS was high. Approximately half of MGUS patients already had HTN, DM, HLD, and OA at the time of MGUS diagnosis, and these comorbidities occurred in approximately half of the remaining MGUS patients during the follow-up period.

The prevalence of MGUS in those older than 50 years has been reported to be 3.6–3.9% in Western countries^10,25^ and 0.8–3.3% in Eastern countries^26–28^. In this study, the prevalence rate per 100,000 was 0.37–0.50 overall and 1.11–1.52 in those aged 50 years or older in South Korea between January 1, 2007, and August 31, 2009. Considering that the world standardized incidence rate for MGUS was 3.76 ± 0.26 per 100,000 inhabitants reported in a population-based study in France^29^, the prevalence of MGUS in South Korea between January 1, 2007, and August 31, 2009, is thought to be relatively low compared to other countries. However, it is difficult to directly compare the prevalence of previous studies with those of the present study. In this study, we calculated the period prevalence by dividing the total number of patients with MGUS by the mid-year population. In other words, this study determined prevalence using the claim data for MGUS as a numerator and the mid-year population as the denominator, which is an arbitrary number representing the population. During the recruitment period, asymptomatic cases or cases in which the individual did not receive treatment could not be captured by this method; thus, the prevalence of MGUS could be somewhat underreported. In addition, since the mid-year population could be larger than the actual population, there is a possibility that the prevalence of MGUS in this study was somewhat underreported. In contrast, previous studies conducted screening tests for M-protein in all subjects regardless of symptoms, and these studies were conducted in limited participant groups recruited in community practices, hospitals, or regions, rather than the entire population.

It should be noted that the number of patients with MGUS is increasing every year compared to the patient registration period of this study (January 1, 2007, to August 31, 2009). This trend has not only been seen in South Korea, but also in other Asian countries, including Japan, Taiwan, and Hong Kong^27,28,30^. The reasons for this increase could be related to rapid industrialization and increased life span, which are common trends among Asian countries. Since aging is associated with the incidence of MGUS, increased life span may have increased the prevalence of MGUS. Alternatively, as interest in MGUS and diseases related to MGUS increases, more cases are assessed for MGUS, which may lead to greater detection of MGUS cases. Additionally, the prevalence of MGUS decreased in those over 80 years of age in this study, contrary to what it has been widely demonstrated in previous studies that indicated that prevalence of MGUS increases with increasing age^10,25^. Considering that this study was based on analysis of insurance claims data, it is possible that people in this age group had more passive hospital visits and treatment compared to younger people. Alternatively, this trend may be due to limited access to medical services in older patients.

In this study, a total of 158 of 470 patients with MGUS (33.62%) progressed to MM (28.51%), PCL (0.43%), extramedullary plasmacytoma (0.64%), solitary plasmacytoma (0.43%), WM (1.49%), and amyloidosis (2.13%) during a 10-year follow-up period. MM, WM, and amyloidosis showed a tendency to occur within 2 years after the initial diagnosis of MGUS; in contrast, PCL, extramedullary plasmacytoma, and solitary plasmacytoma occurred sporadically throughout the follow-up period. The molecular basis of MGUS progression to symptomatic monoclonal gammopathy remains poorly understood; however, several genetic aberrations may be involved in its mechanism. Taking this into account, the genetic factors involved in the mechanism of progression of symptomatic monoclonal gammopathy may differ from each other, which may have led to the above epidemiological pattern^3,31–34^. The 10-year cumulative probability of progression to MM in this study was higher than previously known (approximately 10%)^1,7,18,35^, which may also be due to racial and genetic differences. Alternatively, it could be due to insufficient work-up to exclude symptomatic monoclonal gammopathy at the time of diagnosis of MGUS in the real world because routine bone marrow examination or CT in all patients with MGUS was a controversial guideline in 2010^4,6,7,36^. In fact, in the present study, bone marrow examination, spinal CT, and spinal MRI were performed in only 20.2%, 1.5%, and 0.9% of 470 patients with MGUS, respectively. In addition, approximately 50% of patients progressed to MM within 3 months from the date of MGUS diagnosis. Based on the results of this study, it can be suggested that when diagnosing patients with MGUS, sufficient tests should be performed to differentiate symptomatic monoclonal gammopathy, especially MM. If sufficient testing is not performed, close follow-up within three months of diagnosis is necessary. Another important consideration is that since this study selected patients with MGUS based on insurance claims data, patients without symptoms or specific clinical abnormalities may have been excluded. Therefore, the higher 10-year cumulative probability of progression reported in this study may have been due to the under-diagnosis of low-risk cases of MGUS with a low risk of disease progression.

Interestingly, approximately 30–50% of patients with MGUS had HTN, DM, HLD, and OA at the time of diagnosis of MGUS, and these comorbidities were newly developed during the follow-up period in approximately 50% of patients who did not have any comorbidities at the time of diagnosis of MGUS. According to data from the Korean National Health and Nutrition Examination Survey, the prevalence of HTN, DM, HLD, and OA in the population aged 50 years and older in South Korea is reported to be approximately 35%, 15%, 50%, and 35%, respectively^37–41^. Although direct comparison is impossible, it is deemed a significant number, considering that out of the total 470 patients with MGUS, the probability of already having or someday having these comorbidities is up to 80%. This may be because MGUS might be associated with the pathophysiology of HTN, DM, HLD, and OA. Alternatively, it may have been found that the chances of visiting the hospital and undergoing tests increased during the follow-up period for MGUS, considering the high incidence of these comorbidities within 5 years of MGUS diagnosis (Fig. 3). Conversely, a population that has already been diagnosed with HTN, DM, HLD, OA or belonging to a high-risk group that could proceed to these conditions have higher healthcare utilization and are therefore more likely to be tested for MGUS, that may have led to the results of this study.

Monitoring for kidney disease in patients with MGUS is important because it is one of the end-organ damages that can be caused by disease progression. In contrast, monoclonal gammopathy of renal significance (MGRS), in which one or more kidney lesions related to the produced monoclonal immunoglobulin occur without disease progression^42^. In this study, approximately 30% of 470 patients with MGUS had existing or newly developed acute or chronic kidney disease. This is a fairly high number compared to the pooled incidence of acute kidney disease in the general hospitalized population, which is 19.4% in Eastern Asia according to KDIGO-equivalent criteria^43^. The total prevalence estimate of chronic kidney disease in adults aged over 20 years in South Korea was 8.2%^44^. Supplementary Figures 3 and 4 present cases of newly occurring acute or chronic kidney disease in patients with MGUS divided according to disease progression. In this study, 15/470 patients (3.19%) and 13/470 patients (2.77%) were diagnosed with acute or chronic kidney disease, respectively, several months before disease progression. Additionally, 52/470 patients (11.06%) and 63/470 patients (13.40%) were diagnosed with acute or chronic kidney disease, respectively, without evidence of disease progression. These patients may have had MGRS. MGRS-associated kidney diseases do not respond well to the immunosuppressive regimens, and may necessitate clone-directed therapy. Affected patients have an approximately 90% recurrence after kidney transplantation if monoclonal gammopathy is not eliminated before or immediately after transplantation^45–47^. Therefore, it is necessary to raise awareness for these patients and actively consider kidney biopsy if MGRS is suspected.

In addition, approximately 20–40% of all MGUS patients in this study had pre-existing or newly diagnosed comorbidities such as thyroid disorders, disc disorders, peripheral neuropathy, myocardial infarction, stroke, and heart failure. These prevalence and incidence rates are higher than previously known^48–54^. However, it is difficult to conclude whether MGUS or underlying comorbidities may be risk factors for these diseases. Nevertheless, according to the results of this study, it is considered necessary to monitor not only disease progression but also the comorbidities mentioned above in the follow-up of patients with MGUS. In the case of solid malignancies, less than 5% of patients with MGUS were newly identified within the follow-up period. This is similar to the previously known average risk^55–59^; therefore, it is reasonable to perform cancer screening in MGUS patients in the same manner as in the general population.

This study analyzed claims data using the HIRA database; therefore, the present study has several limitations. First, because information on risk factors, including individual patient's type of MGUS and blood test at the time of diagnosis cannot be identified, detailed risk groups cannot be classified. Second, it was impossible to directly analyze the risk compared to the matched general population because HIRA only provided data concerning the population with MGUS claims data rather than the entire population (HIRA policy due to database capacity limitations). For example, this study showed that the prevalence of the aforementioned comorbidities in patients with MGUS was higher than the prevalence data for each comorbidity in the entire population provided by the public HIRA database (2010). However, it was not possible to determine whether this was due to MGUS or other combined comorbidities, as the comparison was not made with the general population, which was corrected for several factors that could influence these results. Third, we defined the prevalence of comorbidities at diagnosis of MGUS as comorbidities from January 1, 2007, to the date of diagnosis with MGUS, and newly developed comorbidities were defined as newly developed diseases in MGUS patients who did not have any comorbidities at the time of diagnosis of MGUS. There is a limitation in that the follow-up period, according to the date of diagnosis of MGUS, differed in determining the prevalence of comorbidities or the number of newly developed cases in this study. Although the distribution of the date of diagnosis with MGUS was relatively even except for January 2007, and the median follow-up duration for the definition of prevalence was 15.61 months, which was close to the average patient enrollment period (Supplementary Figure 5), it is necessary to be cautious in interpreting the prevalence of comorbidities or newly developed diseases presented in this study. Nevertheless, it is considered to be important because this study presented the prevalence of MGUS in South Korea and information on the occurrence patterns of disease progression and comorbidities during a 10-year follow-up period of patients with MGUS in the real world.

In conclusion, in this study, 33.62% of the patients with MGUS experienced disease progression to symptomatic monoclonal gammopathies during a 10-year follow-up in the real world. Most patients had MM, and the incidence rate was high within 2 years of the diagnosis of MGUS. In addition, approximately 80% of patients with MGUS were diagnosed with HTN, DM, HLD, and OA at the time of diagnosis or during the follow-up period. Taken together, when MGUS is diagnosed, close follow-up of the possibility of progression to MM is required, especially within 2 years after diagnosis; at the same time, various comorbidities should be considered and monitored during the follow-up of patients with MGUS, especially HTN, DM, HLD, and OA. Since this study was performed by analyzing insurance claims data, it must be taken into account that the prevalence of MGUS and its correlative comorbidities could have been underestimated from the actual prevalence, and more studies are needed to transition from the current disease progression detection-centered guidelines to those that can improve overall patient care.

## Supplementary Information

Supplementary Information.
