# Supplementary material for: A nationwide study of patients with monoclonal gammopathy of undetermined significance with a 10-year follow-up in South Korea
Source: Sci Rep. 2021 Sep 16;11:18449. doi: 10.1038/s41598-021-97664-y (PMC8445957; doi:10.1038/s41598-021-97664-y)

A nationwide study of patients with monoclonal gammopathy of undetermined significance with a 10-year follow-up in South Korea

Ka-Won Kang[1], Ji Eun Song[2], Byung-Hyun Lee[1], Min Ji Jeon[1], Eun Sang Yu[1], Dae Sik Kim[1], Se Ryeon Lee[1], Hwa Jung Sung[1], Chul Won Choi[1], Yong Park[1], and Byung Soo Kim[1]

[1]Division of Hematology-Oncology, Department of Internal Medicine, Korea University College of Medicine, Seoul, South Korea

[2]Department of Biostatistics, Korea University College of Medicine, Seoul, South Korea

Supplementary Table 1. Patients with MGUS analyzed in this study

Subject	Patients (n)
**Included patients**	
D472 Patients who were diagnosed with MGUS as main diagnosis or sub-diagnosis from January 1, 2007 to August 31, 2009	643
**Excluded patients***	
C90.0 Multiple myeloma	128
C90.1 Plasma cell leukemia	1
C90.2 Extramedullary plasmacytoma	10
C90.3 Solitary plasmacytoma	0
C88.0 Waldenström macroglobulinemia	7
E85.3, E85.4, E85.8, E85.9 Amyloidosis†	14
C81 Hodgkin lymphoma‡	1
C82 Follicular lymphoma‡	1
C83 Non-follicular lymphoma‡	3
C84 Mature T/NK-cell lymphomas‡	1
C85 Other and unspecified types of non-Hodgkin lymphoma‡	8
C86 Other specified types of T/NK-cell lymphoma‡	0
C88.4 Extranodal marginal zone B-cell lymphoma of mucosa-associated lymphoid tissue [MALT-lymphoma]	0
C91 Lymphoid leukemia‡	0
C92 Myeloid leukemia‡	3
C93 Monocytic leukemia‡	0
C94 Other leukemias of specified cell type‡	1
C95 leukemia of unspecified cell type‡	3
C96 Other and unspecified malignant neoplasms of lymphoid, hematopoietic and related tissue‡	0
Total excluded patients	173∫
**Total number of patients analyzed in this study**	**470**

Note: Disease classification was performed according to the Korea Classification of Disease (7th edition) disease classification code. * From January 1, 2007 to the date of diagnosis of MGUS, all patients with the relevant codes were excluded. † Only secondary amyloidosis was selected for exclusion. ‡ This entry includes all sub-codes. ∫ Eight patients had the relevant codes in duplicate.

Supplementary Table 2. Mid-year population according to Korean Statistical Information Service survey results

Year	Sex	Total	0-9 years	10-19 years	20-29 years	30-39 years	40-49 years	50-59 years	60-69 years	70-79 years	80-89 years	90-99 years	≥100 years
**2007**	**Total**	50,144,604	5,360,693	6,782,559	7,404,699	8,714,448	8,498,413	5,701,710	3,789,399	2,145,193	1,669,317	76,105	2,071
	**Male**	24,909,095	2,791,124	3,589,335	3,812,687	4,431,071	4,333,766	2,851,682	1,769,337	832,057	482,118	15,705	216
	**Female**	25,235,509	2,569,569	3,193,224	3,592,012	4,283,377	4,164,648	2,850,028	2,020,062	1,313,137	1,187,200	60,400	1,855
**2008**	**Total**	50,498,196	5,183,839	6,817,497	7,273,832	8,570,849	8,602,965	5,991,650	3,909,750	2,269,326	1,794,749	81,501	2,242
	**Male**	25,063,053	2,695,506	3,605,985	3,751,232	4,366,438	4,377,870	3,002,131	1,837,226	893,794	515,639	16,991	243
	**Female**	25,435,144	2,488,333	3,211,512	3,522,600	4,204,411	4,225,095	2,989,519	2,072,524	1,375,532	1,279,110	64,511	1,999
**2009**	**Total**	50,833,594	5,009,916	6,818,286	7,126,950	8,420,914	8,700,672	6,324,325	4,006,144	2,405,240	1,931,423	87,259	2,467
	**Male**	25,205,712	2,600,690	3,603,701	3,682,227	4,295,222	4,424,693	3,170,224	1,894,727	961,242	554,120	18,560	308
	**Female**	25,627,882	2,409,226	3,214,585	3,444,724	4,125,692	4,275,979	3,154,102	2,111,417	1,443,998	1,377,303	68,699	2,159
**2010**	**Total**	51,140,935	4,838,515	6,810,007	6,927,509	8,324,779	8,715,639	6,726,716	4,092,725	2,536,642	2,071,222	94,546	2,638
	**Male**	25,329,453	2,507,717	3,594,307	3,590,734	4,244,774	4,434,091	3,369,420	1,946,828	1,027,907	592,782	20,510	385
	**Female**	25,811,482	2,330,798	3,215,701	3,336,775	4,080,006	4,281,548	3,357,296	2,145,897	1,508,735	1,478,440	74,036	2,253

Note: This data is summarized by extracting information provided on the official website of the Korean Statistical Information Service (http://kosis.kr/statisticsList/statisticsListIndex.do?menuId=M_01_01&vwcd=MT_ZTITLE&parmTabId=M_01_01#SelectStatsBoxDiv).

Supplementary Figure 1. The prevalence calculation method of each comorbidity in the entire population

$$\text{Overall prevalence} = \frac{\text{The total number of patients diagnosed with the corresponding disease during the year}}{\text{Mid-year population}} \times 100$$

$$\text{The prevalence among those aged over 50 years} = \frac{\text{The number of patients >50 years diagnosed with the corresponding disease during the year}}{\text{Mid-year population >50 years}} \times 100$$

Supplementary Figure 2. The duration from the date of diagnosis of MGUS to the date of diagnosis of MM in this study

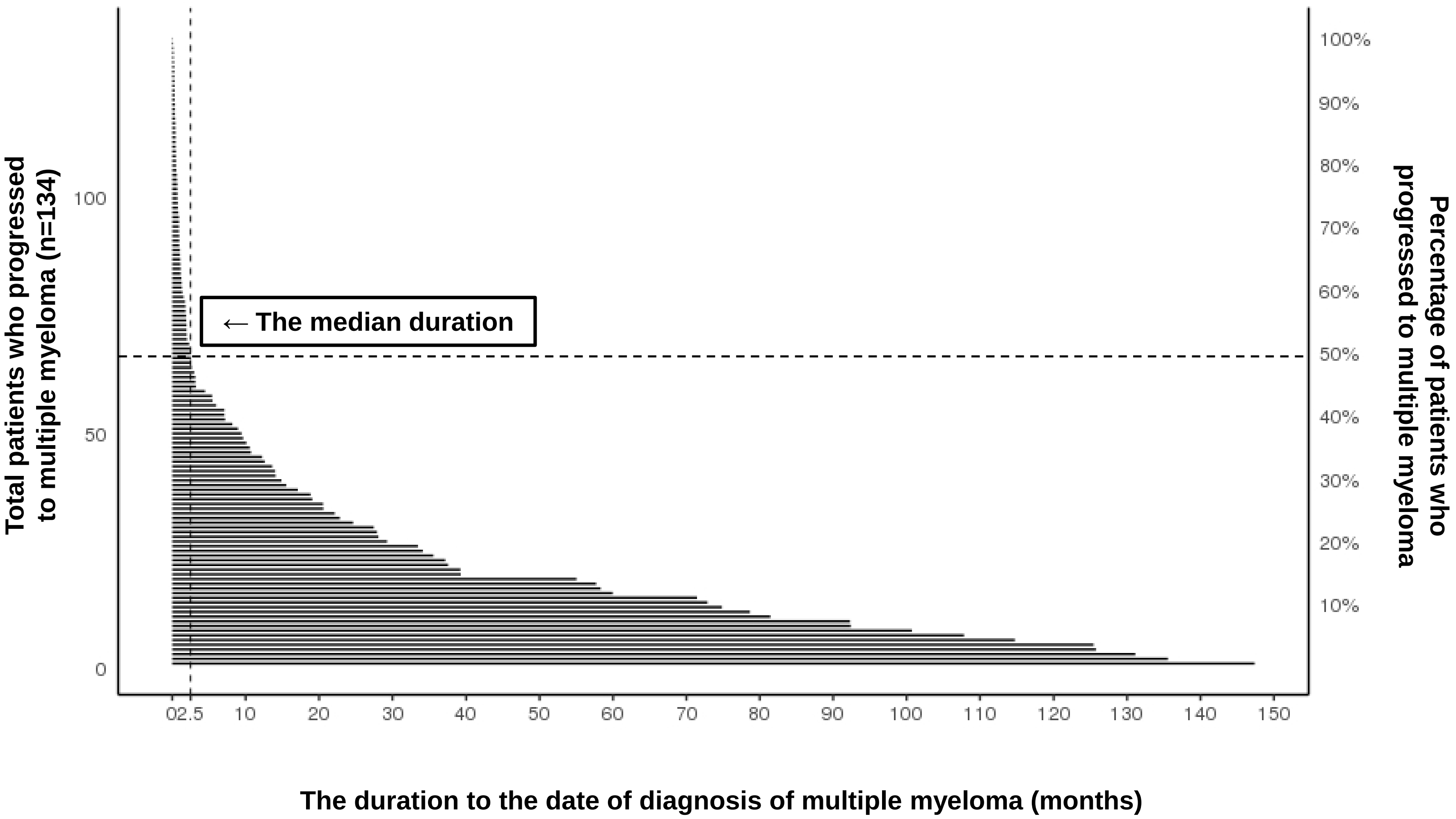

Supplementary Figure 3. Newly occurring acute or chronic kidney disease in patients with MGUS who progressed to symptomatic monoclonal gammopathy (n=158)

Note: Claims data for acute or chronic kidney disease; each patient is represented as a data point.

Acute kidney disease

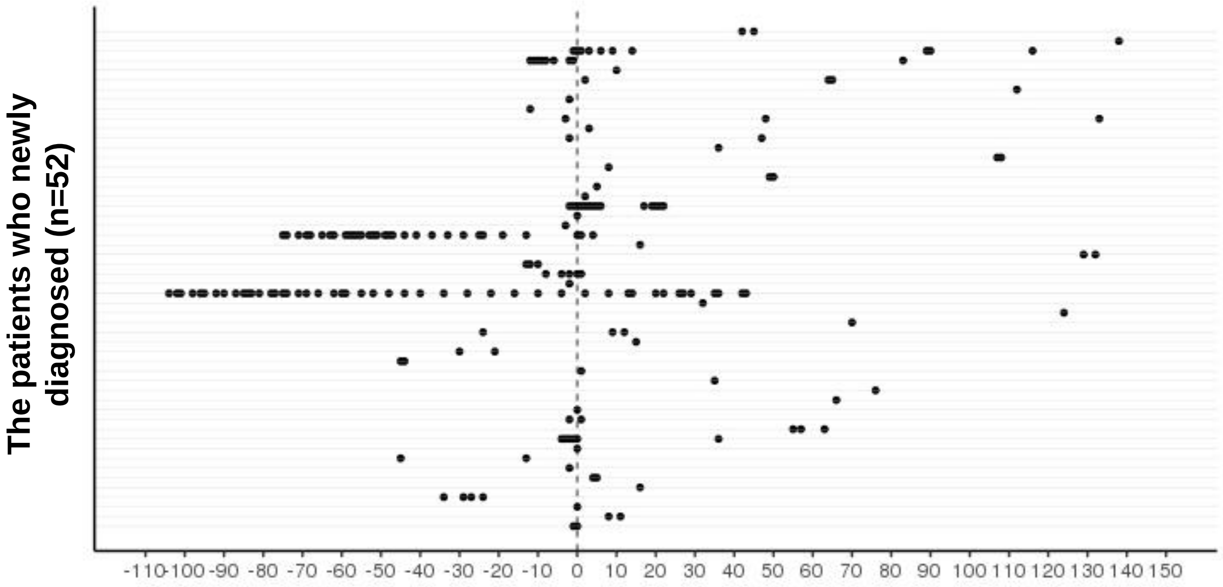

Before progression (- months)	Time of disease progression diagnosis	After progression (+ months)
15 patients (28.8%)	11 patients (21.2%)	26 patients (50%)

Chronic kidney disease

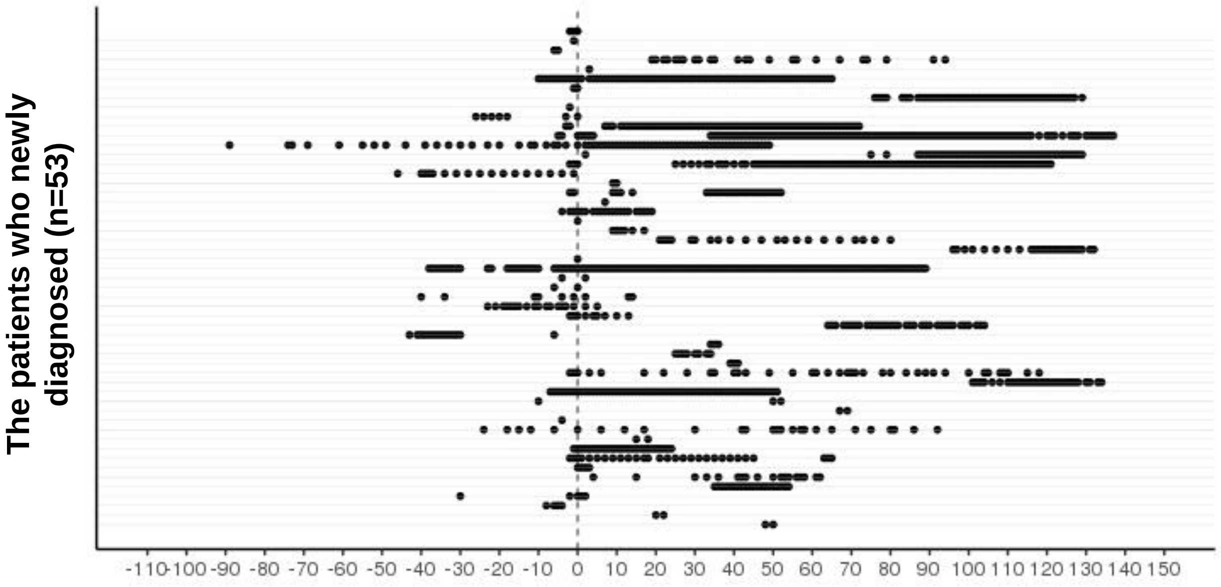

Before progression (- months)	Time of disease progression diagnosis	After progression (+ months)
13 patients (24.6%)	20 patients (37.7%)	20 patients (37.7%)

Supplementary Figure 4. Newly occurring acute or chronic kidney disease in patients with MGUS who did not progress to symptomatic monoclonal gammopathy (n=312)

Note: The incidence and prevalence of newly developed acute or chronic kidney disease were presented as the number of new patients during the year, and total patients during the year, respectively.

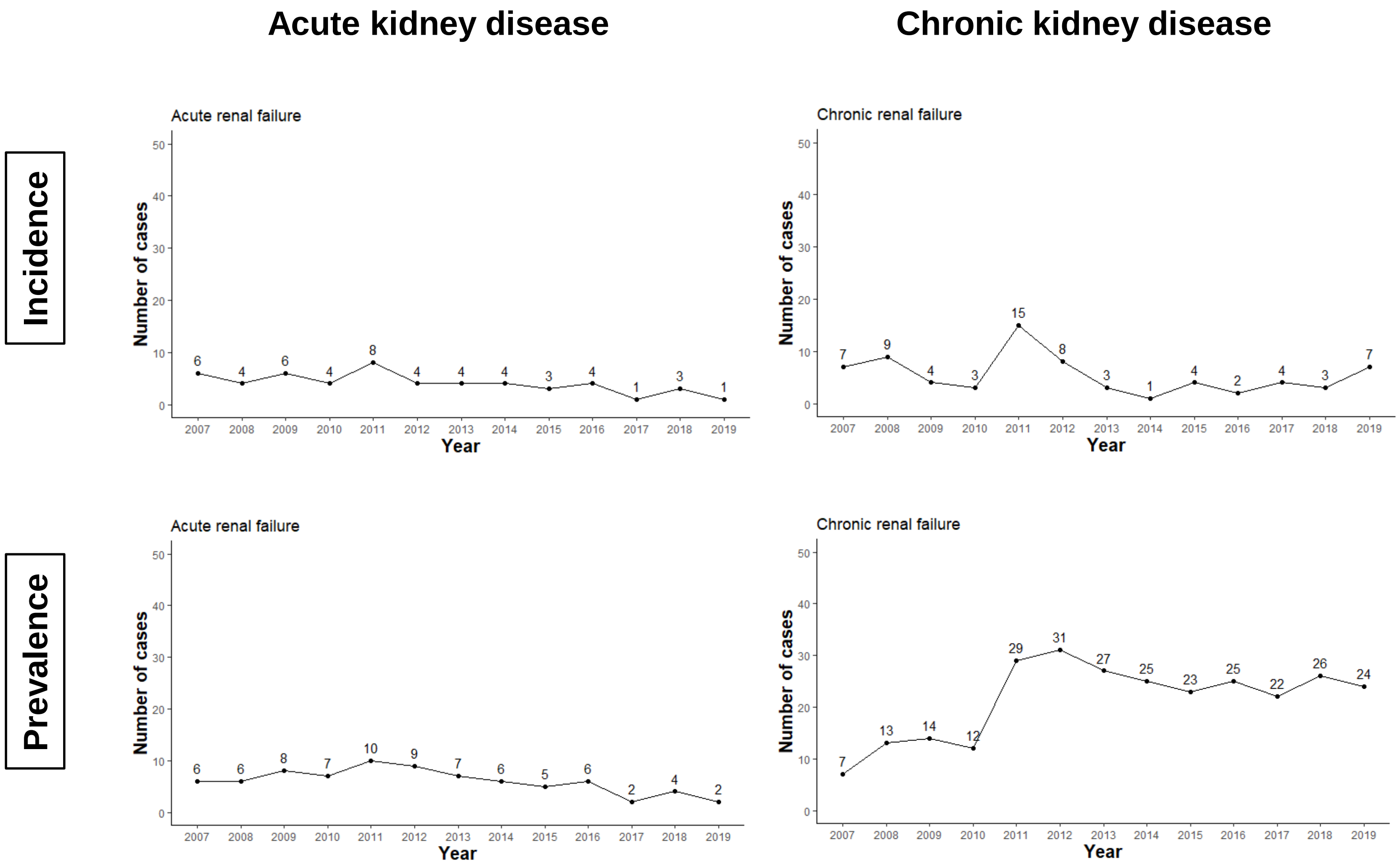

In total, 52 patients (16.7%) were newly diagnosed with acute kidney disease during the follow-up period

In total, 63 patients (20.2%) were newly diagnosed with chronic kidney disease during the follow-up period

Supplementary Figure 5. Distribution of the date of MGUS diagnosis in this study

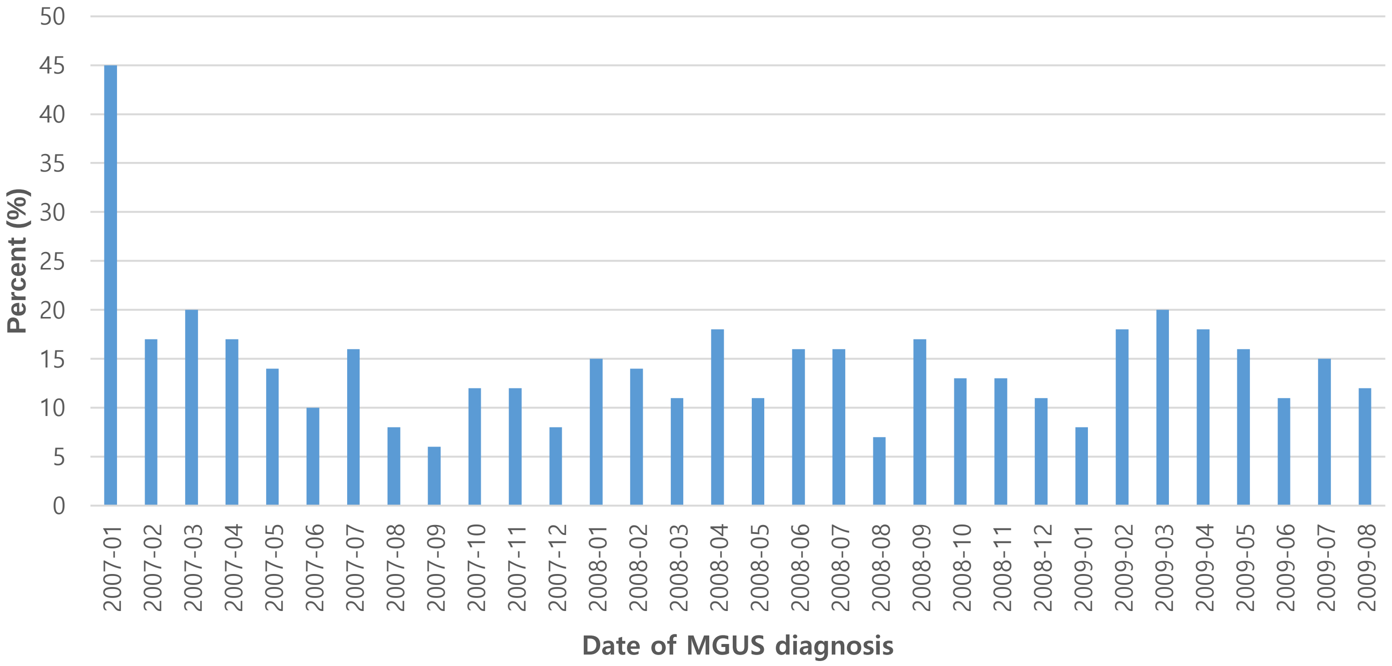

Supplement: Supplementary file 1 — Supplementary Information. [file 41598_2021_97664_MOESM1_ESM.pdf]
